# Psychoacoustically guided midfrequency band-limiting improves the diagnostic utility of classical acoustic measures in dysphonia

**DOI:** 10.1038/s41598-026-44010-9

**Published:** 2026-03-15

**Authors:** Kiyohito Hosokawa, Itsuki Kitayama, Shinobu Iwaki, Misao Yoshida, Akira Miyauchi, Kenji Aruga, Takanari Kawabe, Toshihiro Kishikawa, Hidenori Tanaka, Takeshi Tsuda, Yoshiyuki Ozono, Yukinori Takenaka, Makoto Ogawa, Hidenori Inohara

**Affiliations:** 1 Department of Otorhinolaryngology and Head and Neck Surgery, The University of Osaka Graduate School of Medicine, 2-2 Yamadaoka, Suita-City, Osaka 565-0871 Japan; 2 Department of Otorhinolaryngology, Osaka International Medical and Science Center, Osaka, Japan; 3 Department of Rehabilitation, Kobe University Hospital, Kobe, Hyogo Japan; 4 Department of Rehabilitation, Sakai Heisei Hospital, Osaka, Japan; 5 Department of Surgery, Kuma Hospital, Kobe, Hyogo Japan

**Keywords:** Acoustic analysis, Dysphonia, Psychoacoustics, Perturbation measures, Voice quality, Engineering, Health care, Medical research, Neuroscience

## Abstract

Human hearing is most sensitive around 3 kHz, where small changes in harmonic structure and noise are particularly noticeable. However, traditional or standard acoustic measures of dysphonia—jitter, shimmer, harmonics-to-noise ratio, and cepstral peak prominence smoothed—are usually calculated from full-band signals and do not account for this psychoacoustic weighting. Consequently, their diagnostic accuracy is often reduced in connected speech and cases of mild dysphonia, which are common clinical presentations. We tested a simple, low-cost preprocessing strategy that limited analysis to the 2–4 kHz frequency band before computing these measures. Using sustained vowels and connected speech from Japanese speakers across a wide range of dysphonia severities, we compared band-limited and full-band results against the auditory-perceptual judgments of breathiness, roughness, and the grade of hoarseness. Across utterance and perceptual dimensions, band-limiting consistently improved diagnostic performance, with particularly notable gains in mild dysphonia. These findings suggest that restricting analysis to the high-sensitivity auditory range reveals microperturbations that are masked by dominant low-frequency energy in full-band signals. This psychoacoustically guided approach enhances the clinical usefulness of traditional acoustic measures without requiring complex modeling. It is device-independent and suitable for telemedicine and real-world voice-assessment workflows.

**Supplementary Information:**

The online version contains supplementary material available at 10.1038/s41598-026-44010-9.

## Introduction

Voice quality assessment is essential for diagnosing and managing dysphonia^1–4^. In clinical practice, auditory–perceptual tools, such as the Grade, Roughness, Breathiness, Asthenia, and Strain (GRBAS) scale^5^ and the Consensus Auditory–Perceptual Evaluation of Voice ^6,7^, are widely used. However, their reliability is inherently limited by inter- and intrarater variability, required training, and vulnerability to contextual biases^8–11^. To supplement these perceptual methods, quantitative acoustic measures have been developed to quantify vocal fold vibration irregularities and noise-related features of the glottal source. Traditional perturbation metrics, including jitter, shimmer, and the harmonics-to-noise ratio (HNR), are mainly suitable for sustained vowels^12–15^. In contrast, cepstral measures such as the cepstral peak prominence (CPP) and cepstral peak prominence smoothed (CPPS) can be applied to sustained vowels and connected speech and are now central to modern acoustic assessment^16,17^. Despite this, these measures often show reduced diagnostic accuracy for connected speech and mild dysphonia, highlighting the difficulty of aligning acoustic metrics with human perception^18–22^. These limitations motivate the development of alternative acoustic approaches that are more sensitive to subtle deviations and less affected by speech-related variability.

This gap has motivated frequency-dependent approaches to dysphonia assessment, including studies examining how spectral filtering alters conventional perturbation and noise-related measures^23^ and multi-band analyses that derive band-wise features from sustained vowels and connected speech^24,25^. In addition, auditory-inspired filterbank spectral analyses have been used to characterize how dysphonic and typical voices differ across frequency bands and task types (sustained vowels vs running speech), suggesting that dysphonia-related information can vary by both spectral region and phonation context^26^. Relatedly, band-energy–based spectral measures (e.g., mean energy in the 1–3 kHz region) have been used to quantify frequency-specific energy redistribution associated with speech production changes such as increased vocal effort and clear/communicative speech adaptations^27–29^. While these approaches share the general premise that perceptually relevant information can be frequency-dependent, they treat band energy itself as the primary descriptor rather than a preprocessing step. These prior studies provide important context, but they primarily focus on band-wise descriptors or on how filtering changes the measures themselves; in contrast, the present study asks whether restricting the analyzed signal to an auditory-salient band before computing classical acoustic measures can improve their alignment with auditory–perceptual ratings.

Human auditory sensitivity varies substantially across frequencies and is greatest in the middle-kHz range, where perceptual thresholds are lowest. This heightened sensitivity, shaped by outer-ear resonance and cochlear mechanics, makes midfrequency spectral and temporal fluctuations particularly salient^30^.

In this study, we introduce a psychoacoustically guided signal-conditioning strategy to address this perceptual–acoustic mismatch. Specifically, we restricted the analysis to the 2–4 kHz band—aligned with peak auditory sensitivity—before computing classical acoustic measures. We hypothesized that this band-limiting approach would enhance perceptually relevant microperturbations, particularly in mild dysphonia and connected speech, where conventional measures often underperform. Using a large corpus of Japanese sustained vowels and connected speech, we compared jitter, shimmer, HNR, and CPPS derived from full-band and band-limited signals against GRBAS ratings. For connected speech, only voiced segments were analyzed. These were automatically detected and concatenated using the Acoustic Breathiness Index (ABI)-based procedure^31^, so that feature extraction was performed exclusively on voiced intervals from the standardized reading passage. This voiced-only processing has been used in prior work to improve the robustness of perturbation and cepstral measures in connected-speech recordings by reducing the influence of unvoiced and transitional segments^31,32^. Diagnostic performance was evaluated using receiver operating characteristic (ROC) analysis and the area under the ROC curve (AUC), and association patterns were examined using rank-based correlation, to characterize when band-limiting is beneficial and when it is not.

## Results

Table 1 summarizes the diagnostic performance, expressed as AUC, for each acoustic measure in sustained vowels and connected speech. Notably, improvements in discrimination performance (AUC) were not always accompanied by higher absolute correlations with perceptual scores, and in some measure–material–dimension combinations, band-limiting increased AUC while reducing the absolute Spearman’s correlation coefficient.

**Table 1:** Diagnostic performance of acoustic measures derived from full-band and 2–4 kHz band-limited signals.

Task	Score	Measure	Full-band voice ROC					Band-limited voice ROC					Comparison	
			AUC (95% confidence interval)	Cutoff	Sensitivity	Specificity	N	AUC (95% confidence interval)	Cutoff	Sensitivity	Specificity	N	*p* value	N
Sustained vowel	G	Jitter	0.832 (0.779–0.885)	0.346	0.821	0.744	454	**0.899 (0.868–0.930)**	0.528	0.911	0.828	445	**0.003**	444
		Shimmer	0.851 (0.810–0.893)	3.227	0.911	0.691	454	**0.891 (0.856–0.927)**	13.440	0.911	0.789	444	**0.042**	443
		HNR	0.865 (0.823–0.907)	21.952	0.875	0.746	454	**0.901 (0.869–0.934)**	9.712	0.857	0.851	445	**0.037**	444
		CPPS	0.835 (0.795–0.875)	15.604	0.946	0.667	455	**0.872 (0.836–0.909)**	8.673	0.821	0.805	455	**0.003**	455
	B	Jitter	0.817 (0.767–0.868)	0.346	0.772	0.768	454	**0.893 (0.861–0.925)**	0.546	0.861	0.85	445	**0.000**	444
		Shimmer	0.847 (0.806–0.889)	2.831	0.823	0.771	454	0.881 (0.847–0.915)	14.364	0.911	0.773	444	0.076	443
		HNR	0.852 (0.809–0.894)	21.952	0.823	0.773	454	**0.884 (0.850–0.919)**	7.749	0.861	0.781	445	**0.025**	444
		CPPS	0.849 (0.812–0.886)	15.604	0.924	0.699	455	**0.879 (0.847–0.912)**	8.593	0.797	0.827	455	**0.006**	455
	R	Jitter	**0.743 (0.697–0.788)**	0.47	0.78	0.618	454	0.691 (0.639–0.742)	0.754	0.568	0.744	445	**0.008**	444
		Shimmer	**0.751 (0.706–0.796)**	5.163	0.907	0.526	454	0.639 (0.584–0.693)	15.518	0.547	0.679	444	**0.000**	443
		HNR	**0.780 (0.737–0.823)**	19.231	0.853	0.625	454	0.688 (0.635–0.741)	5.327	0.682	0.63	445	**0.000**	444
		CPPS	0.647 (0.596–0.697)	12.401	0.893	0.38	455	0.641 (0.587–0.695)	6.832	0.647	0.626	455	0.653	455
Connected speech	G	Jitter	0.686 (0.627–0.746)	1.815	0.733	0.569	453	**0.809 (0.764–0.853)**	2.881	0.86	0.628	449	**0.000**	448
		Shimmer	0.651 (0.592–0.711)	10.169	0.977	0.256	453	**0.796 (0.745–0.848)**	16.615	0.767	0.725	449	**0.000**	448
		HNR	0.619 (0.560–0.678)	12.533	0.965	0.264	454	**0.817 (0.775–0.859)**	5.086	0.872	0.663	451	**0.000**	450
		CPPS	0.824 (0.785–0.863)	12.903	0.907	0.669	455	0.786 (0.738–0.835)	13.950	0.791	0.678	455	0.100	455
	B	Jitter	0.681 (0.631–0.731)	1.889	0.72	0.574	453	**0.842 (0.805–0.879)**	3.407	0.872	0.663	449	**0.000**	448
		Shimmer	0.638 (0.588–0.689)	10.09	0.933	0.308	453	**0.810 (0.767–0.853)**	17.036	0.726	0.772	449	**0.000**	448
		HNR	0.609 (0.558–0.661)	12.608	0.909	0.324	454	**0.860 (0.826–0.894)**	5.086	0.811	0.774	451	**0.000**	450
		CPPS	**0.892 (0.862–0.922)**	12.895	0.866	0.797	455	0.809 (0.768–0.849)	13.973	0.695	0.777	455	**0.000**	455
	R	Jitter	0.754 (0.710–0.798)	1.94	0.793	0.587	453	0.744 (0.699–0.789)	3.315	0.753	0.629	449	0.636	448
		Shimmer	0.772 (0.729–0.814)	7.874	0.755	0.643	453	0.730 (0.683–0.776)	19.314	0.824	0.524	449	0.084	448
		HNR	0.771 (0.728–0.813)	15.193	0.788	0.611	454	0.748 (0.703–0.793)	4.158	0.83	0.558	451	0.265	450
		CPPS	0.747 (0.703–0.791)	11.765	0.842	0.601	455	0.729 (0.683–0.775)	13.677	0.788	0.579	455	0.400	455

Values represent area under the receiver operating characteristic curve (AUC).

*p* values indicate significance of differences between full-band and band-limited AUCs based on DeLong’s test.

Bold values indicate statistically significant differences (*p* < 0.05).

AUC, area under the receiver operating characteristic curve; G, Grade; B, Breathiness; R, Roughness; HNR, harmonics-to-noise ratio; CPPS, cepstral peak prominence smoothed.

**Fig. 1:**
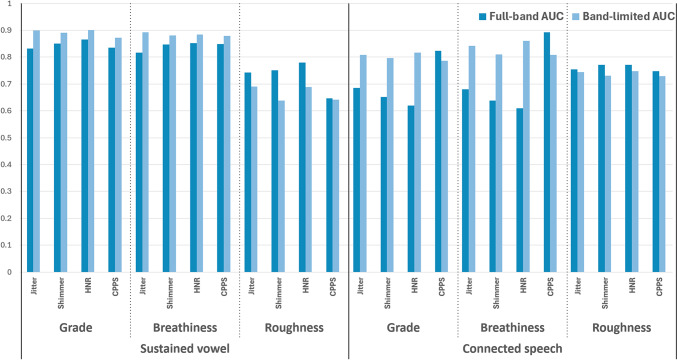
Diagnostic performance (AUC) of the full-band and 2–4 kHz band-limited signals.

Table 2 presents the corresponding Spearman’s correlation coefficients with the GRBAS scale. Figure 1 graphically displays the AUC values from Table 1 and shows a consistent improvement when using 2–4 kHz band-limited signals, particularly for the Grade and Breathiness components. The following subsections detail these findings according to task and perceptual dimension. Descriptive statistics for each acoustic measure (full-band and 2–4 kHz band-limited) are provided in Supplementary Table S1. The AUC and correlation results for mildly dysphonic voices in each perceptual dimension are described in Tables 3 and 4, respectively.

**Table 2:** Spearman’s correlation coefficients between acoustic measures and GRBAS ratings for full-band and 2–4 kHz band-limited signals.

Task	Score	Measure	Full-band			Band-limited			Comparison	
			Correlation	*p* value	N	Correlation	*p* value	N	*p* value	N
Sustained vowel	G	Jitter	0.750	0.000	454	**0.806**	0.000	445	**0.009**	444
		Shimmer	**0.793**	0.000	454	0.725	0.000	444	**0.043**	443
		HNR	− 0.818	0.000	454	− 0.787	0.000	445	0.379	444
		CPPS	**− 0.830**	0.000	455	− 0.787	0.000	455	**0.013**	455
	B	Jitter	0.716	0.000	454	**0.789**	0.000	445	**0.002**	444
		Shimmer	0.778	0.000	454	0.717	0.000	444	0.080	443
		HNR	− 0.796	0.000	454	− 0.765	0.000	445	0.402	444
		CPPS	**− 0.820**	0.000	455	− 0.772	0.000	455	**0.007**	455
	R	Jitter	**0.592**	0.000	454	0.497	0.000	445	**0.002**	444
		Shimmer	**0.628**	0.000	454	0.388	0.000	444	**0.000**	443
		HNR	**− 0.666**	0.000	454	− 0.483	0.000	445	**0.000**	444
		CPPS	**− 0.470**	0.000	455	− 0.421	0.000	455	**0.011**	455
Connected speech	G	Jitter	0.515	0.000	453	**0.732**	0.000	449	**0.000**	448
		Shimmer	0.496	0.000	453	**0.630**	0.000	449	**0.000**	448
		HNR	− 0.460	0.000	454	**− 0.743**	0.000	451	**0.000**	450
		CPPS	**− 0.809**	0.000	455	− 0.628	0.000	455	**0.000**	455
	B	Jitter	0.422	0.000	453	**0.717**	0.000	449	**0.000**	448
		Shimmer	0.400	0.000	453	**0.622**	0.000	449	**0.000**	448
		HNR	− 0.341	0.000	454	**− 0.725**	0.000	451	**0.000**	450
		CPPS	**− 0.790**	0.000	455	− 0.599	0.000	455	**0.000**	455
	R	Jitter	0.596	0.000	453	0.547	0.000	449	0.187	448
		Shimmer	**0.620**	0.000	453	0.469	0.000	449	**0.000**	448
		HNR	**− 0.624**	0.000	454	− 0.563	0.000	451	**0.049**	450
		CPPS	**− 0.600**	0.000	455	− 0.514	0.000	455	**0.011**	455

Significant values are in bold.

G, G score; B, B score; R, R score; HNR, harmonics-to-noise ratio; CPPS, cepstral peak prominence smoothed.

**Table 3:** Diagnostic performance of acoustic measures derived from full-band and 2–4 kHz band-limited signals in mildly dysphonic voices.

Task	Score	Measure	Full-band voice ROC					Band-limited voice ROC					Comparison	
			AUC (95% confidence interval)	Cutoff	Sensitivity	Specificity	N	AUC (95% confidence interval)	Cutoff	Sensitivity	Specificity	N	*p* value	N
Sustained vowel	G	Jitter	0.728 (0.650–0.805)	0.303	0.696	0.701	260	**0.816 (0.762–0.870)**	0.528	0.911	0.681	260	**0.011**	260
		Shimmer	0.737 (0.669–0.804)	2.831	0.857	0.539	260	**0.819 (0.763–0.875)**	13.440	0.911	0.637	260	**0.015**	260
		HNR	0.767 (0.698–0.836)	24.03	0.714	0.779	260	0.824 (0.769–0.879)	9.712	0.857	0.725	260	0.065	260
		CPPS	0.692 (0.624–0.760)	16.602	0.821	0.554	260	**0.761 (0.698–0.824)**	8.673	0.821	0.632	260	**0.001**	260
	B	Jitter	0.727 (0.659–0.795)	0.346	0.772	0.625	295	**0.828 (0.779–0.877)**	0.546	0.861	0.75	295	**0.001**	295
		Shimmer	0.760 (0.700–0.819)	2.831	0.823	0.62	295	0.814 (0.764–0.864)	14.364	0.911	0.639	295	0.072	295
		HNR	0.764 (0.703–0.826)	23.686	0.684	0.769	295	**0.813 (0.762–0.865)**	8.997	0.797	0.731	295	**0.047**	295
		CPPS	0.744 (0.686–0.802)	16.064	0.861	0.556	295	**0.796 (0.744–0.848)**	8.673	0.785	0.713	295	**0.003**	295
	R	Jitter	**0.662 (0.606–0.718)**	0.47	0.78	0.495	360	0.615 (0.556–0.675)	0.754	0.568	0.647	355	**0.041**	354
		Shimmer	**0.663 (0.607–0.719)**	4.084	0.8	0.49	360	0.576 (0.516–0.637)	13.644	0.453	0.699	354	**0.001**	353
		HNR	**0.701 (0.647–0.755)**	20.65	0.747	0.595	360	0.620 (0.560–0.679)	8.526	0.493	0.734	355	**0.000**	354
		CPPS	0.543 (0.483–0.602)	12.401	0.893	0.227	361	0.563 (0.503–0.623)	6.832	0.647	0.493	361	0.105	361
Connected speech	G	Jitter	0.577 (0.500–0.653)	1.262	0.256	0.911	266	**0.678 (0.611–0.746)**	2.274	0.709	0.594	266	**0.009**	266
		Shimmer	0.528 (0.452–0.605)	6.762	0.442	0.644	266	**0.698 (0.630–0.765)**	15.078	0.605	0.739	266	**0.000**	266
		HNR	0.508 (0.431–0.584)	13.457	0.174	0.894	266	**0.686 (0.621–0.752)**	6.070	0.733	0.6	266	**0.003**	266
		CPPS	0.669 (0.604–0.735)	12.903	0.907	0.394	266	0.669 (0.601–0.738)	14.272	0.581	0.711	266	1.000	266
	B	Jitter	0.594 (0.531–0.657)	1.879	0.707	0.449	311	**0.753 (0.699–0.808)**	2.274	0.634	0.776	311	**0.000**	311
		Shimmer	0.478 (0.413–0.542)	7.314	0.537	0.503	311	**0.739 (0.683–0.794)**	15.330	0.567	0.844	311	**0.000**	311
		HNR	0.498 (0.433–0.562)	16.025	0.506	0.571	311	**0.775 (0.724–0.827)**	6.070	0.671	0.776	311	**0.000**	311
		CPPS	**0.821 (0.774–0.868)**	13.87	0.713	0.81	311	0.729 (0.674–0.785)	14.264	0.512	0.844	311	**0.001**	311
	R	Jitter	0.675 (0.620–0.729)	1.514	0.44	0.837	368	0.680 (0.626–0.735)	3.315	0.753	0.543	366	0.928	365
		Shimmer	0.691 (0.638–0.745)	6.762	0.505	0.804	368	0.689 (0.635–0.743)	16.277	0.527	0.772	366	0.817	365
		HNR	0.686 (0.632–0.740)	17.75	0.402	0.886	369	0.678 (0.624–0.733)	4.085	0.83	0.446	366	0.696	365
		CPPS	0.657 (0.601–0.713)	11.72	0.848	0.457	370	0.661 (0.606–0.716)	13.675	0.788	0.468	370	0.871	370

Values represent area under the receiver operating characteristic curve (AUC).

*p* values indicate significance of differences between full-band and band-limited AUCs based on DeLong’s test.

Bold values indicate statistically significant differences (*p* < 0.05).

AUC, area under the receiver operating characteristic curve; G, Grade; B, Breathiness; R, Roughness; HNR, harmonics-to-noise ratio; CPPS, cepstral peak prominence smoothed.

**Table 4:** Spearman’s correlation coefficients between acoustic measures and GRBAS ratings for full-band and 2–4 kHz band-limited signals in mildly dysphonic voices.

Task	Score	Measure	Full-band			Band-limited			Comparison	
			Correlation	*p* value	N	Correlation	*p* value	N	*p* value	N
Sustained vowel	G	Jitter	0.444	0.000	260	**0.599**	0.000	260	**0.002**	260
		Shimmer	0.500	0.000	260	0.599	0.000	260	0.059	260
		HNR	− 0.553	0.000	260	− 0.614	0.000	260	0.183	260
		CPPS	− 0.483	0.000	260	**− 0.563**	0.000	260	**0.006**	260
	B	Jitter	0.436	0.000	295	**0.614**	0.000	295	**0.000**	295
		Shimmer	0.526	0.000	295	0.570	0.000	295	0.365	295
		HNR	− 0.545	0.000	295	− 0.600	0.000	295	0.194	295
		CPPS	− 0.531	0.000	295	**− 0.601**	0.000	295	**0.006**	295
	R	Jitter	0.369	0.000	360	0.294	0.000	355	0.057	354
		Shimmer	**0.392**	0.000	360	0.229	0.000	354	**0.000**	353
		HNR	**− 0.449**	0.000	360	− 0.300	0.000	355	**0.000**	354
		CPPS	− 0.185	0.000	361	− 0.211	0.000	361	0.205	361
Connected speech	G	Jitter	0.172	0.005	266	**0.403**	0.000	266	**0.000**	266
		Shimmer	0.061	0.324	266	**0.434**	0.000	266	**0.000**	266
		HNR	− 0.010	0.874	266	**− 0.432**	0.000	266	**0.000**	266
		CPPS	− 0.420	0.000	266	− 0.355	0.000	266	0.251	266
	B	Jitter	0.162	0.004	311	**0.466**	0.000	311	**0.000**	311
		Shimmer	0.062	0.275	311	**0.434**	0.000	311	**0.000**	311
		HNR	− 0.016	0.773	311	**− 0.492**	0.000	311	**0.000**	311
		CPPS	**− 0.574**	0.000	311	− 0.412	0.000	311	**0.001**	311
	R	Jitter	0.380	0.000	368	0.389	0.000	366	0.937	365
		Shimmer	0.389	0.000	368	0.395	0.000	366	0.964	365
		HNR	− 0.385	0.000	369	− 0.387	0.000	366	0.872	365
		CPPS	− 0.369	0.000	370	− 0.346	0.000	370	0.594	370

Significant values are in bold.

G, G score; B, B score; R, R score; HNR, harmonics-to-noise ratio; CPPS, cepstral peak prominence smoothed.

### Signal characteristics after 2–4 kHz band-pass filtering

**Fig. 2:**
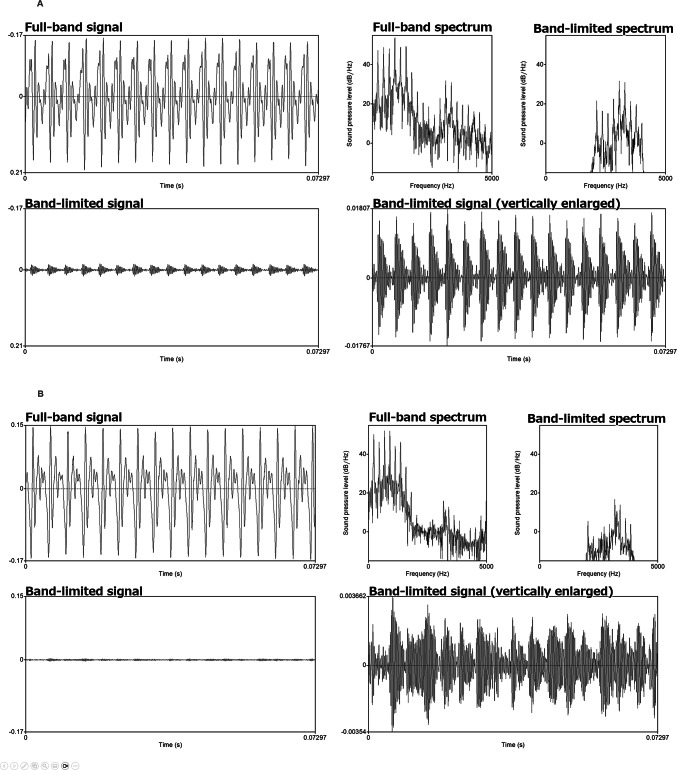
Representative examples of full-band and band-limited signals and spectra. (**A**) Normophonic voice sample. (**B**) Mild breathy voice sample (Breathiness = 0.66). For each sample, the full-band waveform and spectrum are shown together with the band-limited waveform and spectrum; the band-limited waveform is also displayed with an enlarged amplitude scale to visualize temporal fluctuations that are not apparent at the original scale. The band-limited signals were obtained by applying a band-pass filter between 2000 and 4000 Hz.

To characterize the impact of 2–4 kHz band-pass filtering, we compared representative waveforms and spectra before and after filtering (Fig. 2). The band-limited signal showed an approximately ten-fold reduction in waveform amplitude relative to the original full-band signal. In the spectral domain, energy below 2 kHz was strongly attenuated (approximately 26.5 dB), resulting in a relative emphasis of midfrequency components within the 2–4 kHz range.

### Sustained vowels

Overall, applying a 2–4 kHz band-pass filter improved diagnostic performance for Grade and Breathiness, whereas performance for Roughness tended to decrease.

*Grade (G)*: Jitter AUC increased from 0.832 to 0.899 (*p* = 0.0033), and |ρ| also increased from 0.750 to 0.806 (*p* = 0.0088). Shimmer AUC increased from 0.851 to 0.891 (*p* = 0.0417); however, |ρ| decreased from 0.793 to 0.725 (*p* = 0.0426). HNR AUC increased from 0.865 to 0.901 (*p* = 0.0370), whereas |ρ| changed from 0.818 to 0.787 without a significant difference (*p* = 0.3789). CPPS AUC increased from 0.835 to 0.872 (*p* = 0.0027), but |ρ| decreased from 0.830 to 0.787 (*p* = 0.0130).

*Breathiness (B)*: Jitter AUC increased from 0.817 to 0.893 (*p* = 0.0001), and |ρ| increased from 0.716 to 0.789 (*p* = 0.0016). Shimmer AUC changed from 0.847 to 0.881 without a significant difference (*p* = 0.0756), and |ρ| also changed from 0.778 to 0.717 without a significant difference (*p* = 0.0796). HNR AUC increased from 0.852 to 0.884 (*p* = 0.0245), whereas |ρ| changed from 0.796 to 0.765 without a significant difference (*p* = 0.4023). CPPS AUC increased from 0.849 to 0.879 (*p* = 0.0058), and |ρ| decreased from 0.820 to 0.772 (*p* = 0.0068).

*Roughness (R)*: Jitter AUC decreased from 0.743 to 0.691 (*p* = 0.0079), and |ρ| decreased from 0.592 to 0.497 (*p* = 0.0019). Shimmer AUC decreased from 0.751 to 0.639 (*p* < 1e−16), and |ρ| also decreased from 0.628 to 0.388 (*p* < 1e−16). HNR AUC decreased from 0.780 to 0.688 (*p* < 1e−16), and |ρ| decreased from 0.666 to 0.483 (*p* < 1e−16). CPPS AUC changed from 0.647 to 0.641 without a significant difference (*p* = 0.6525), while |ρ| decreased from 0.470 to 0.421 (*p* = 0.0106).

### Mildly dysphonic sustained vowels

In mildly dysphonic sustained vowels, band-limiting to 2–4 kHz improved performance for Grade and Breathiness, whereas Roughness did not show improvement.

*Grade (G)*: Jitter AUC increased from 0.728 to 0.816 (*p* = 0.0109), and |ρ| increased from 0.444 to 0.599 (*p* = 0.0021). Shimmer AUC increased from 0.737 to 0.819 (*p* = 0.0145), whereas |ρ| changed from 0.500 to 0.599 without a significant difference (*p* = 0.0594). HNR AUC changed from 0.767 to 0.824 without a significant difference (*p* = 0.0648), and |ρ| changed from 0.553 to 0.614 without a significant difference (*p* = 0.1826). CPPS AUC increased from 0.692 to 0.761 (*p* = 0.0012), and |ρ| increased from 0.483 to 0.563 (*p* = 0.0057).

*Breathiness (B)*: Jitter AUC increased from 0.727 to 0.828 (*p* = 0.0005), and |ρ| increased from 0.436 to 0.614 (*p* = 0.0001). Shimmer AUC changed from 0.760 to 0.814 without a significant difference (*p* = 0.0715), and |ρ| changed from 0.526 to 0.570 without a significant difference (*p* = 0.3654). HNR AUC increased from 0.764 to 0.813 (*p* = 0.0465), whereas |ρ| changed from 0.545 to 0.600 without a significant difference (*p* = 0.1935). CPPS AUC increased from 0.744 to 0.796 (*p* = 0.0032), and |ρ| increased from 0.531 to 0.601 (*p* = 0.0064).

*Roughness (R)*: Jitter AUC decreased from 0.662 to 0.615 (*p* = 0.0409), while |ρ| changed from 0.369 to 0.294 without a significant difference (*p* = 0.0570). Shimmer AUC decreased from 0.663 to 0.576 (*p* = 0.0005), and |ρ| decreased from 0.392 to 0.229 (*p* = 0.0001). HNR AUC decreased from 0.701 to 0.620 (*p* = 0.0001), and |ρ| decreased from 0.449 to 0.300 (*p* < 1e−16). CPPS AUC changed from 0.543 to 0.563 without a significant difference (*p* = 0.1048), and |ρ| changed from 0.185 to 0.211 without a significant difference (*p* = 0.2046).

### Connected speech

In connected speech, band-limiting improved diagnostic accuracy of perturbation measures for Grade and Breathiness, whereas Roughness did not improve; CPPS tended to decrease for Grade and Breathiness.

*Grade (G)*: Jitter AUC increased from 0.686 to 0.809 (*p* < 1e−16), and |ρ| increased from 0.515 to 0.732 (*p* < 1e−16). Shimmer AUC increased from 0.651 to 0.796 (*p* < 1e−16), and |ρ| increased from 0.496 to 0.630 (*p* = 0.0003). HNR AUC increased from 0.619 to 0.817 (*p* < 1e−16), and |ρ| increased from 0.460 to 0.743 (*p* < 1e−16). CPPS AUC changed from 0.824 to 0.786 without a significant difference (*p* = 0.0996), while |ρ| decreased from 0.809 to 0.628 (*p* < 1e−16).

*Breathiness (B)*: Jitter AUC increased from 0.681 to 0.842 (*p* < 1e−16), and |ρ| increased from 0.422 to 0.717 (*p* < 1e−16). Shimmer AUC increased from 0.638 to 0.810 (*p* < 1e−16), and |ρ| increased from 0.400 to 0.622 (*p* < 1e−16). HNR AUC increased from 0.609 to 0.860 (*p* < 1e−16), and |ρ| increased from 0.341 to 0.725 (*p* < 1e−16). CPPS AUC decreased from 0.892 to 0.809 (*p* < 1e−16), and |ρ| decreased from 0.790 to 0.599 (*p* < 1e−16).

*Roughness (R)*: In connected speech in Roughness, AUCs showed only minimal, non-significant changes after band-limiting (all *p* > 0.05). In contrast, correlations decreased for Shimmer, HNR, and CPPS (*p* = 0.0002, 0.0494, and 0.0110, respectively), while Jitter correlation showed no significant change (*p* = 0.1872).

### Mildly dysphonic connected speech

In mildly dysphonic connected speech, band-limiting improved perturbation measures for Grade and Breathiness, whereas Roughness showed no consistent changes.

*Grade (G)*: Jitter AUC increased from 0.577 to 0.678 (*p* = 0.0086), and |ρ| increased from 0.172 to 0.403 (*p* = 0.0001). Shimmer AUC increased from 0.528 to 0.698 (*p* = 0.0001), and |ρ| increased from 0.061 to 0.434 (*p* < 1e−16). HNR AUC increased from 0.508 to 0.686 (*p* = 0.0033), and |ρ| increased from 0.010 to 0.432 (*p* < 1e−16). CPPS AUC was unchanged (0.669 to 0.669; *p* = 1.000), and |ρ| changed from 0.420 to 0.355 without a significant difference (*p* = 0.2509).

*Breathiness (B)*: Jitter AUC increased from 0.594 to 0.753 (*p* < 1e−16), and |ρ| increased from 0.162 to 0.466 (*p* < 1e−16). Shimmer AUC increased from 0.478 to 0.739 (*p* < 1e−16), and |ρ| increased from 0.062 to 0.434 (*p* < 1e−16). HNR AUC increased from 0.498 to 0.775 (*p* < 1e−16), and |ρ| increased from 0.016 to 0.492 (*p* < 1e−16). CPPS AUC decreased from 0.821 to 0.729 (*p* = 0.0007), and |ρ| decreased from 0.574 to 0.412 (*p* = 0.0005).

*Roughness (R)*: In mildly rough connected speech, band-limiting to 2–4 kHz produced only minimal, non-significant changes; neither AUCs nor correlation coefficients differed significantly between full-band and band-limited conditions (all *p* > 0.05).

## Discussion

This study tested whether restricting acoustic analysis to the 2–4 kHz band can improve agreement between traditional acoustic measures and auditory-perceptual ratings. The impact of band-limiting was strongly dimension- and material-dependent. For Grade and Breathiness, band-limiting often improved discrimination and/or strengthened associations with perceptual severity, particularly for perturbation measures and HNR. For example, in sustained vowels, jitter showed higher AUCs for both Grade (0.832 → 0.899) and Breathiness (0.817 → 0.893), with concurrent increases in |ρ| (Grade: 0.750 → 0.806; Breathiness: 0.716 → 0.789). Similar improvements were observed in connected speech (e.g., Grade jitter AUC 0.686 → 0.809; |ρ| 0.515 → 0.732; Breathiness HNR AUC 0.609 → 0.860; |ρ| 0.341 → 0.725). In contrast, for Roughness, performance decreased in sustained vowels across multiple measures (e.g., shimmer AUC 0.751 → 0.639; |ρ| 0.628 → 0.388; HNR AUC 0.780 → 0.688), and remained largely unchanged in connected speech in terms of AUC, while correlations tended to weaken (e.g., shimmer |ρ| 0.620 → 0.469; CPPS |ρ| 0.600 → 0.514). CPPS showed mixed behavior: it improved AUCs for Grade and Breathiness in sustained vowels (e.g., Grade 0.835 → 0.872; Breathiness 0.849 → 0.879) but did not uniformly strengthen correlations; in connected speech, CPPS decreased for Breathiness (AUC 0.892 → 0.809; |ρ| 0.790 → 0.599). Taken together, psychoacoustically motivated band-limiting can enhance the diagnostic utility of several established measures in contexts dominated by Grade and Breathiness, but it does not confer uniform benefits and may be detrimental for Roughness-related outcomes.

In addition, we note that improvements in discrimination were sometimes accompanied by reduced absolute correlations. This divergence is expected because AUC reflects the separability between predefined groups (e.g., absence vs presence or mild vs absence), whereas correlation quantifies the strength of a monotonic association across an ordinal severity continuum. Band-limiting may enhance group separability by emphasizing midfrequency cues that distinguish categories, while not necessarily preserving the rank order across multiple grades (and vice versa), particularly when severity range is restricted. Importantly, band-limiting extracts only a portion of the speech signal and therefore is not expected to fully match auditory-perceptual judgments, which integrate information across the entire spectrum. Although the 2–4 kHz region is perceptually salient, lower-frequency components typically dominate overall intensity, and both frequency regions can contribute to perceived voice quality. From a psychoacoustic perspective, these findings are consistent with classical models of auditory frequency selectivity and loudness perception. The 2–4 kHz range corresponds to the peak of the equal-loudness contour and closely matches the resonance properties of the outer and middle ear, resulting in lower detection thresholds for amplitude and spectral fluctuations^30,33^. When signals are filtered to this midfrequency band, low-frequency harmonics—normally dominate due to their high amplitudes—are strongly attenuated. As shown in Fig. 2, the filtering substantially attenuates low-frequency energy, which may reduce masking by high-energy low-frequency components and increase the relative prominence of midfrequency features. By suppressing these energetically dominant low-frequency components, the filtering process effectively unmasks higher-order harmonics and turbulence-related noise in the 2–4 kHz range. These midfrequency elements become more pronounced relative to the reduced low-frequency energy, thereby increasing the perceptual and computational salience of microperturbations. Consequently, perturbation metrics such as jitter and shimmer may become more sensitive to cycle-to-cycle irregularities that are otherwise obscured in the full-band signal^30^. This psychoacoustic mechanism may help explain the observed improvements in discriminability and the higher absolute correlations with perceptual ratings of Grade and Breathiness.

In contrast, Roughness did not benefit from band-limiting and showed consistent decreases in both the AUC and absolute correlation. This divergence highlights the multidimensional nature of dysphonia and the different acoustic mechanisms underlying each perceptual dimension. Roughness is mainly driven by low-frequency amplitude modulations, subharmonics, period doubling, and other nonlinear vibratory phenomena^30,31,34–41^. These features primarily occur below 2 kHz and are therefore attenuated or removed by the present filtering strategy. The current results support the view that roughness depends on low-frequency and broadband information and cannot be reliably quantified through high-frequency-focused perturbation or cepstral measures. This interpretation is consistent with the multivariate definition of the Acoustic Roughness Index, which incorporates subharmonic energy and low-frequency modulation cues to achieve higher predictive accuracy for roughness^38,39^. Accordingly, the present filtering strategy should be viewed as complementary rather than substitutive, and roughness quantification likely requires low-frequency and/or broadband information.

*Technical considerations*: Restricting the signal to the 2–4 kHz band should not be interpreted as “purifying” the voice signal or yielding a cleaner estimate of overall voice quality. Rather, band-pass filtering changes the spectral balance and may therefore affect the meaning and robustness of classical acoustic measures. First, attenuating low-frequency energy can reduce the prominence of the source-related harmonic structure, which may influence the stability of perturbation estimates (e.g., jitter and shimmer) depending on the implementation and the extent to which reliable periodicity is preserved within the retained band. Notably, even when the fundamental component is strongly attenuated, periodicity cues can still be carried by the remaining harmonic structure within the passband (i.e., a “missing fundamental” situation), as illustrated in Fig. 2. Accordingly, perturbation measures computed from the band-limited signal should be interpreted as reflecting irregularities in the retained mid-frequency harmonic components rather than direct estimates of glottal period or amplitude perturbations. Second, because higher-frequency components often include turbulence-related noise, excluding these frequencies may alter the contribution of aperiodic energy and can bias noise-sensitive measures. Third, HNR is inherently defined by the relative balance between periodic and aperiodic components; therefore, HNR computed from band-limited signals may not be directly comparable to full-band HNR with respect to global voice quality. For these reasons, the present approach should be viewed as complementary to conventional full-band analyses rather than a substitute, and its utility is expected to be dimension- and context-dependent.

*Clinical implications*: The present findings suggest that 2–4 kHz band-limiting may serve as a complementary preprocessing step for acoustic analyses when the goal is to improve discrimination for Grade and Breathiness in specific contexts. This approach is not intended to alter routine auditory-perceptual evaluation; perceptual ratings should be performed on the unfiltered signal, which preserves the full spectral information relevant to clinical listening and to perceptual dimensions such as roughness. In future work, band-limited features may be integrated into multivariate acoustic indices (e.g., ABI-like composites) and/or used as a component of automated screening and classification workflows. Because dysphonia etiologies differ in their spectral signatures, the benefit of 2–4 kHz band-limiting is expected to vary across pathologies and voice qualities. In particular, conditions in which audible breathiness is driven by increased glottal leakage and turbulence-related noise may show larger gains for Breathiness-related discrimination, whereas disorders dominated by low-frequency nonlinear phenomena may benefit less from a midfrequency-focused approach. Importantly, the present study did not optimize filtering by diagnosis, and future work should evaluate performance stratified by etiology and voice phenotype to clarify generalizability and define when band-limited features add the most clinical value.

*Limitations*: First, although 2–4 kHz aligns with the established peak of human auditory sensitivity, the optimal frequency band may differ across languages, vocal tasks, and listeners. Crosslinguistic differences in spectral balance and vowel quality could change the contribution of midfrequency harmonics, so future work should test generalizability across languages. Second, because recordings were made under controlled acoustic conditions, further studies are needed to confirm robustness in noisy environments and across diverse recording devices. Third, this study applied a fixed 2–4 kHz band-limiting approach; more refined frequency-weighting strategies using scaled auditory filters^40^ or modulation filter banks may yield additional improvements^37^. Limitation related to mild-subgroup analyses. Because the mild-dysphonia analyses were restricted to a narrow severity range (0 < score ≤ 1 on the GRBAS-based reference score), these subgroup analyses are subject to range restriction, which reduces variance in perceptual ratings. This restriction can attenuate or otherwise distort association metrics such as Spearman’s rank correlation, particularly when the underlying perceptual scale is an ordinal 0–3 structure. Therefore, correlation results within the mild-subgroup should be interpreted cautiously, and the mild-subgroup analyses are presented primarily to evaluate discrimination performance under subtle-deviation conditions rather than to characterize associations across the full severity spectrum.

In conclusion, incorporating psychoacoustic principles into the preprocessing stage of acoustic voice analysis improves the diagnostic performance of classical perturbation and cepstral measures for grading hoarseness and breathiness in both sustained vowels and connected speech. By emphasizing the 2–4 kHz range, band-limiting can improve discriminability for Grade and Breathiness in specific contexts, while association patterns are measure- and task-dependent and may not uniformly increase across conditions. This simple, device-independent approach offers a practical refinement for current voice-assessment pipelines and may support more reliable dysphonia screening in clinical and remote healthcare settings.

## Methods

### Voice dataset

This study used the same voice dataset as our previous investigations, comprising 455 voice recordings from Japanese speakers^38,41^. The dataset included sustained vowels and connected speech from adults with a wide range of organic and functional voice disorders, as well as normophonic controls. Participant demographics (age, sex) and clinical characteristics (diagnosis) are summarized in Supplementary Table S2, together with the distribution of perceptual grades (0–3) for Grade, Roughness, and Breathiness. Detailed inclusion and exclusion criteria, diagnostic categories, and treatment histories have been reported in the original studies^38,41^. Demographic information (sex, age, and diagnosis) and auditory–perceptual ratings of Grade, Roughness, and Breathiness were available for both sustained vowels and connected speech. Ratings were made by three trained raters with confirmed intra- and inter-rater reliability in the previous study^41^. Each participant produced a sustained vowel /a/ and read a standardized Japanese translation of “The North Wind and the Sun” at a comfortable pitch and loudness in a sound-treated room. Recordings were digitized at a 44.1-kHz sampling rate and 16-bit resolution using an omnidirectional head-mounted condenser microphone (SE50, Samson Technologies Corp.) and an external digital recorder (H4n linear PCM recorder, Zoom Corp.).

### Auditory-perceptual evaluation

Voice quality was rated using the GRBAS scale, in which Grade, Roughness, and Breathiness are scored on a 4-point ordinal scale (0 = normal, 1 = mild, 2 = moderate, 3 = severe). Auditory-perceptual rating data used in the present study were obtained from our previously published dataset^41^. In that dataset, three trained raters had been selected as reliable raters based on predefined reliability criteria (Cohen’s κ > 0.40 for pairwise agreement and Fleiss’ κ > 0.40 for overall agreement), and their ratings were used to construct the reference perceptual scores. For the present analyses, a single reference score for each dimension was obtained by averaging the three rater scores (range 0–3 in 1/3-point steps). Inter- and intra-rater reliability for this rater panel and dataset are summarized in Supplementary Table S3. Based on commonly used guidelines for κ statistics, both Cohen’s κ (pairwise agreement) and Fleiss’ κ (three-rater agreement) exceeded 0.40, suggesting at least moderate agreement for the present ratings.

For subgroup analyses focusing on mild dysphonia, “mild” was operationally defined per perceptual dimension as a reference score in the interval 0 < score ≤ 1. Recordings with score = 0 were treated as absence of that perceptual attribute for that dimension, and recordings with score > 1 were excluded from the mild-subgroup analyses. For descriptive purposes, severity categories were defined based on the reference perceptual score as absent = 0, mild = 0 < score ≤ 1, moderate = 1 < score ≤ 2, and severe = 2 < score ≤ 3 (Supplementary Table S2).

### Signal preprocessing

**Fig. 3:**
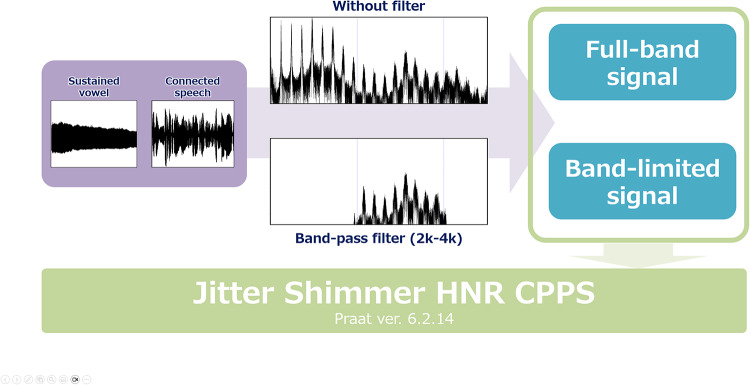
Signal-processing pipeline for full-band and band-limited analysis.

Figure 3 presents an overview of the preprocessing pipeline, illustrating the signal flow from the original sustained vowel and connected speech recordings to the full-band waveforms and the 2–4 kHz band-limited signals used for acoustic analysis.

All signal processing and acoustic measurements were conducted in *Praat* (version 6.2.14). For sustained vowels, the middle 3 s of each /a/ phonation were extracted, excluding onset and offset portions. For connected speech, only voiced segments were analyzed. These were automatically detected and concatenated using the ABI algorithm^31^, ensuring that analyses included only voiced intervals from the standardized reading passage. This approach has been shown to substantially improve the stability and diagnostic performance of perturbation and cepstral measures—such as jitter, shimmer, the HNR, and CPPS—by minimizing the confounding influence of unvoiced and transitional segments^32,42^.

Each recording was analyzed in two forms: (1) the original full-band signal and (2) a 2–4 kHz band-limited version. The band-limited signals were generated in *Praat* using a Hann band-pass filter with the command: Filter (pass Hann band): 2000, 4000, 100, applying 100-Hz smoothing at the filter edges. All acoustic measures were computed in parallel for both signal types.

To demonstrate the effect of band-pass filtering, waveforms and spectra were visually inspected before and after filtering (Fig. 2). Although the filter removed harmonic components below 2 kHz, including the fundamental frequency and lower harmonics, the temporal periodicity associated with the fundamental frequency remained visible in the filtered waveform. This preserved periodic structure allowed reliable computation of perturbation measures (jitter, shimmer, HNR) and CPPS even in the absence of low-frequency harmonic energy. Spectrally, the filtering emphasized higher-order harmonics and turbulence-related noise in the midfrequency range, supporting the psychoacoustic rationale for this preprocessing strategy.

### Acoustic measurements

Jitter, shimmer, and HNR were calculated using the same *Praat* script settings as in the ABI framework. For each waveform, the following sequence was applied:

- To Pitch (cc)… 0 75 15 no 0.03 0.45 0.01 0.35 0.14 600
- To PointProcess (cc)
- voiceReport$ = Voice report… 0 0 70 600 1.3 1.6 0.03 0.45
- jitter = 100*extractNumber (voiceReport$, "Jitter (local): ")
- shimmer = 100*extractNumber (voiceReport$, "Shimmer (local): ")
- hnr = extractNumber (voiceReport$, "Mean harmonics-to-noise ratio: ")

Jitter (local) and shimmer (local) were expressed as percentages, and HNR was expressed in dB. The same script was applied to both full-band and 2–4 kHz band-limited signals.

For CPPS, we followed the ABI *Praat* implementation using:

- To PowerCepstrogram… 60 0.002 5000 50
- cpps = Get CPPS… no 0.01 0.001 60 330 0.05 Parabolic 0.001 0 Straight Robust

This procedure used a pitch floor of 60 dB, a time step of 0.002 s, a maximum frequency of 5000 Hz, and a quefrency range of 0.01–0.001 s with robust fitting, consistent with ABI scripts. CPPS was computed separately for the full-band and band-limited signals. No additional pre-analysis amplitude normalization was performed before calculating those acoustic measures. Perturbation measures were computed using identical analysis settings for the full-band and 2–4 kHz band-limited signals. Although the filter attenuates low-frequency energy, periodicity cues remain in the harmonic structure within the passband (Fig. 2), allowing cycle-related metrics to be computed consistently across conditions.

### Statistical analysis

Spearman’s rank correlation coefficients between acoustic measures (jitter, shimmer, HNR, and CPPS) and perceptual scores (Grade, Breathiness, and Roughness) were calculated using R (version 4.4.1). Because some measures (e.g., HNR and CPPS) negatively correlate with dysphonia severity, changes in the association strength between full-band and band-limited conditions were assessed by comparing absolute correlation values, noting whether they increased or decreased.

Diagnostic performance for each acoustic measure and perceptual dimension was evaluated using ROC analysis. For ROC/AUC analyses, binary endpoints (absent vs present) were defined per perceptual dimension using the reference GRBAS-based score (score = 0 vs > 0). Thus, “present” indicates the presence of the respective perceptual attribute (Grade, Breathiness, or Roughness) for that dimension, irrespective of the other dimensions. In addition, subgroup ROC analyses were conducted for mildly dysphonic sustained vowels. “Mild” was operationally defined per perceptual dimension as 0 < score ≤ 1 based on the reference score; recordings with score = 0 and recordings with score > 1 were excluded from the mild-subgroup analyses.

AUC values were computed for both full-band and 2–4 kHz band-limited conditions. Comparisons of AUCs between full-band and 2–4 kHz band-limited signals were performed in R (version 4.4.1) using the *pROC* package. For each pair of ROC curves from the same sample set (e.g., full-band jitter vs. band-limited jitter), DeLong’s test for two correlated ROC curves assessed differences in AUC. All tests used a two-sided significance level of *p* < 0.05.

The relationships between diagnostic scores and acoustic parameters were assessed using Spearman’s rank correlation coefficient. Due to the presence of tied ranks in the dataset, *p* values were determined using asymptotic approximation. Differences between dependent Spearman correlation coefficients were evaluated using Steiger’s Z-test. For all analyses, a *p* value of less than 0.05 was considered statistically significant.

### Ethics approval

The study protocol was approved by the Institutional Review Boards of The University of Osaka (15497), Osaka International Medical & Science Center (568), and Kuma Hospital (20120614-1). The study was conducted in accordance with the 1964 Declaration of Helsinki and its later amendments. Given the retrospective nature of the study, the requirement for written informed consent was waived by the ethics committees, as permitted by the ethical guidelines for medical and health research involving human subjects issued by the Ministry of Health, Labour and Welfare of Japan.

## Supplementary Information

Below is the link to the electronic supplementary material.

Supplementary Material 1

Supplementary Material 2

Supplementary Material 3

## Data Availability

Data supporting the results of this study are available in the text. Recorded audio samples are available for research purposes from the corresponding author upon reasonable request.
